# Sprifermin: Effects on Cartilage Homeostasis and Therapeutic Prospects in Cartilage-Related Diseases

**DOI:** 10.3389/fcell.2021.786546

**Published:** 2021-12-14

**Authors:** Zongmian Song, Yusheng Li, Chunfeng Shang, Guowei Shang, Hongwei Kou, Jinfeng Li, Songfeng Chen, Hongjian Liu

**Affiliations:** ^1^ Department of Orthopedics, The First Affiliated Hospital of Zhengzhou University, Zhengzhou, China; ^2^ Department of Orthopedics, Xiangya Hospital, Central South University, Changsha, China

**Keywords:** sprifermin, FGF18, cartilage homeostasis, disease modifying osteoarthritis drugs, osteoarthritis, cartilage-related diseases

## Abstract

When suffering from osteoarthritis (OA), articular cartilage homeostasis is out of balance and the living quality declines. The treatment of knee OA has always been an unsolved problem in the world. At present, symptomatic treatment is mainly adopted for OA. Drug therapy is mainly used to relieve pain symptoms, but often accompanied with adverse reactions; surgical treatment involves the problem of poor integration between the repaired or transplanted tissues and the natural cartilage, leading to the failure of repair. Biotherapy which aims to promote cartilage *in situ* regeneration and to restore endochondral homeostasis is expected to be an effective method for the prevention and treatment of OA. Disease-modifying osteoarthritis drugs (DMOADs) are intended for targeted treatment of OA. The DMOADs prevent excessive destruction of articular cartilage through anti-catabolism and stimulate tissue regeneration via excitoanabolic effects. Sprifermin (recombinant human FGF18, rhFGF18) is an effective DMOAD, which can not only promote the proliferation of articular chondrocyte and the synthesis of extracellular matrix, increase the thickness of cartilage in a dose-dependent manner, but also inhibit the activity of proteolytic enzymes and remarkedly slow down the degeneration of cartilage. This paper reviews the unique advantages of Sprifermin in repairing cartilage injury and improving cartilage homeostasis, aiming to provide an important strategy for the effective prevention and treatment of cartilage injury-related diseases.

## Introduction

The pathogenesis of OA mostly involves the whole joint, mainly including cartilage, subchondral bone, joint capsule, synovial membrane and surrounding muscles, most manifested as cartilage injury, subchondral osteosclerosis and synovial inflammation (Karsdal et al., 2016; Mehana et al., 2019; Wu et al., 2020). Cartilage, a kind of connective tissue without nerves and blood vessels, exists in joint, intervertebral disc, ear, nose, etc., and plays an important role in the normal structural composition of human joint and movement (Krishnan and Grodzinsky, 2018; Vincent and Wann, 2019; Żylińska et al., 2021). Articular cartilage is mainly composed of chondrocytes and extracellular matrix (ECM), the ECM mainly contains water, type II collagen (COL2), proteoglycan and glycosaminoglycan (GAG) (Muller et al., 2020). Cartilage is extremely vulnerable to overloading, inflammation, trauma, etc., and articular cartilage damage is one of the major pathological changes of OA.

Articular cartilage has a poor ability to repair itself due to inadequate blood supply and low metabolism. The cartilage in femorotibial joint can relieve pressure and maintain frictionless movement of joint. If the injury of cartilage in OA is not repaired in time, it will further aggravate and involve the surrounding tissues (Martínez-Moreno et al., 2019; Zhao et al., 2019; Zhang S. et al., 2020). Results of microscopy studies showed that the cartilage structure is disorganized, the number of chondrocytes decreased, and the synthesis of COL2, proteoglycan and GAG is obviously reduced during the occurrence of OA, and eventually lead to cartilage tear. The ulcerated surface is easily formed following cartilage tear, which can be covered by connective tissue or fibrocartilage, accompanied by neovascularization, resulting in damage of articular cartilage with the loss of its full-thickness and damage to the biomechanics of articular cartilage (Brody, 2015; McCulloch et al., 2019; Chen et al., 2020). With the further progression of OA, more matrix degradation related enzymes, such as matrix metalloproteinase-13 (MMP-13) and metalloproteinase-5 (ADAMTS-5), inflammatory factors such as tumor necrosis factor α (TNFα) and interleukin-1β (IL-1β) are produced, eventually leading to deteriorative biochemical changes in articular cartilage (van den Berg, 2011; Malemud, 2019; Pu et al., 2021; Zhang et al., 2021). The above biomechanical and biochemical changes ignite each other, forming a “positive feedback” effect similar to a vicious cycle, synergistically disturbing cartilage homeostasis, aggravating the damage of cartilage and its surrounding tissues, eventually accelerating the development of OA (Kapoor et al., 2011; Varady and Grodzinsky, 2016).

Symptomatic treatment is mainly adopted for knee OA, but lacks effective targeted treatment options (Katz et al., 2021). The treatment plan mainly includes: 1. Drug intervention, such as oral acetaminophen, non-steroidal anti-inflammatory drugs, glucosamine, chondroitin sulfate and intraarticular injection of sodium hyaluronate (Alexander et al., 2020; Wolff et al., 2021). 2. Exercise, proper exercise helps strengthen muscle strength and improves joint function (Skou et al., 2018). 3. Weight management, encouraging overweight patients to lose weight, thereby reducing the pressure load and inflammation state in the joints (Springer et al., 2017; Harasymowicz et al., 2020; Oliveira et al., 2020). 4. Traditional Chinese medicine treatment, such as Chinese medicine ointment, massage, acupuncture and moxibustion, which are mainly used to improve blood circulation (Walsh et al., 2017; Zhang Z. et al., 2020; Park et al., 2021). 5. Surgical treatment, such as microfracture surgery, arthroscopic debridement, unicompartmental knee arthroplasty (UKA), total knee arthroplasty, etc. (Deng et al., 2021; Katz et al., 2021) (Table 1). Conservative treatment is mainly used to relieve pain symptoms, but cannot effectively prevent or reverse the progression of OA (Hochberg et al., 2012). There are also some shortcomings in surgical treatment, such as postoperative prosthesis infection, prosthesis loosening, and limited prosthesis life (not suitable for young patients) (Quinn et al., 2018; Xu et al., 2019). Relevant techniques for promoting cartilage repair through cell or cartilage transplantation have once attracted the attention of many scholars (Lv et al., 2020). However, most of these technologies have the problem of poor integration between the repaired or transplanted tissue and the natural cartilage, which changes the stress distribution during the joint load and causes the repaired or transplanted tissues to degrade, ultimately leading to failure of transplantation repair (Li Z. et al., 2016; Ernstbrunner et al., 2018). If biological therapy can effectively promote cartilage repair *in situ* and restore cartilage homeostasis, it will provide a powerful means for the effective prevention and treatment of OA. Disease-modifying osteoarthritis drugs (DMOADs) are a class of drugs that can be used to treat OA. DMOADs mainly include fibroblast growth factor 18 (FGF18), bone morphogenetic protein-7 (BMP-7), C-type natriuretic peptide (CNP), insulin like growth factor-1 (IGF-1) etc. (Davies et al., 2008; Hunter et al., 2010; Bükülmez et al., 2014; Zhang et al., 2017; Muller et al., 2020; Shah and Mithoefer, 2020b). (Table2). Sprifermin (recombinant human FGF18, rhFGF18) is an effective DMOAD. In the literature Sprifermin is the only DMOAD which can strongly and effectively maintain the chondrocyte phenotype in cell culture models (Gigout et al., 2017; Antunes et al., 2020; Muller et al., 2020). Sprifermin markedly promotes the proliferation of articular chondrocytes and the synthesis of ECM, and thus increases cartilage thickness in a dose-dependent manner (Hochberg et al., 2019). Also, it can efficiently inhibit proteolytic enzyme activity (such as MMP-13 and ADAMTS-5) and significantly reduce articular cartilage degeneration (Hochberg et al., 2018; Hochberg et al., 2019; Muller et al., 2020). Sprifermin is currently in phase III clinical trial, and no local or systemic safety concerns have been reported (Mori et al., 2014; Li et al., 2021; Zeng et al., 2021). In view of this, we reviewed the unique advantages of Sprifermin in promoting chondrocyte proliferation and ECM synthesis, repairing articular cartilage injury and improving cartilage homeostasis, as well as analyzed its possible molecular mechanisms, aiming to provide an important guidance for the effective prevention and treatment of articular cartilage injury related diseases with OA as a typical example.

**TABLE 1:** Treatment options for OA.

Treatment characteristics	
Drug intervention	Oral or intravenous medication can temporarily relieve symptoms, but long-term use does not enhance the efficacy and increases toxic side effects
	Compared with oral or intravenous medication, topical drugs have less side effects, but skin allergies may occur
	Articular injection is a common clinical treatment method, but it has the risk of infection
Exercise	Enhance muscle strength, increase joint stability, and maintain joint mobility
Weight management	Reduce the pressure load and inflammation state in the joints
Surgical treatment	Surgery can effectively relieve symptoms and improve joint function, however, surgery has risks such as postoperative prosthesis infection, prosthesis loosening and limited prosthesis life
Traditional Chinese medicine treatment	Improve blood circulation, relieve pain, alleviate swelling and improve articular function
Cell or cartilage transplant	Cell or cartilage transplantation is an emerging treatment. The surgical incision is small, and the postoperative recovery is quick, but there is the possibility of poor integration of the repaired joint
DMOADs	Promote articular chondrocyte proliferation and synthesis of ECM, inhibit proteolytic enzyme activity, repair the damaged cartilage, and improve cartilage homeostasis, prevent or reverse the progress of OA

**TABLE 2:** Comparison of various DMOADs.

DMOADs	Characteristics	References
Sprifermin	Sprifermin is the only compound favoring the chondrocyte phenotype, and it does not increase the expression of any hypertrophy markers. Sprifermin can reduce type I collagen in ECM. Compared with permanent exposure, intermittent exposure can maximize Sprifermin’s anabolic potential. Sprifermin has better effect on promoting chondrocyte proliferation relative to several other DMOADs	Eckstein et al. (2021)
BMP-7	BMP-7, also known as osteogenic protein-1, is a growth factor in normal articular cartilage, and there it shows similar results between intermittent and permanent exposure. BMP-7 can stimulate cartilage differentiation and promote the synthesis and retention of ECM. BMP-7, like Sprifermin, has entered clinical trials	Hunter et al. (2010)
CNP	Compared with other DMOADs, CNP has weaker promotion effect on chondrocyte proliferation and ECM production	Bükülmez et al. (2014)
IGF-1	IGF-1 is better at promoting chondrocytes proliferation during permanent exposure, and it can regulate transcription of degrading enzymes to inhibit the rate of matrix degradation, but IGF-1 is found to increase COL1 expression during permanent exposure	Zhang et al. (2017)
TGF-β1	TGF-β1 (transforming growth factor-β1) can induce SOX-9 expression in MSC (mesenchymal stem cell) and increase cartilaginous ECM. TGF-β1 can promote chondrocytes to form cartilage-like tissue	Davies et al. (2008)

## Basic Structure and Function of Sprifermin

The fibroblast growth factor (FGF) family is a group of proteins with homology in nuclear acid sequences, which plays an important role in many pathophysiological processes such as embryo development, cell growth, tissue repair, tumor growth and invasion etc. (Hung et al., 2016; Khosravi et al., 2021; Liu et al., 2021). There are currently 19 members in the FGF family. In the late 1990s, FGF18 was identified as a new member of the FGF family (Hu et al., 1998). FGF18 was firstly isolated from mouse embryos by Maruoka et al. (Hu et al., 1998; Maruoka et al., 1998). Structurally, it is similar to FGF8 and FGF17 and is a member of the same subfamily, with 70–80% amino acid homology sequence. FGF18 is a highly conserved protein composed of 207 amino acids, and the gene encoding this protein is located on chromosome 5q34 (Whitmore et al., 2000). Yang et al. confirmed that FGF18 overexpression effectively inhibits the epithelial-mesenchymal transformation of renal clear cell carcinoma through PI3K/Akt signaling pathway (Yang et al., 2020). Boylan et al. documented that FGF8, FGF17 and FGF18 are crucial for the formation of the fetal abdominal wall (Boylan et al., 2020). Current studies in the field of OA have reported that FGF18 remarkedly stimulates the proliferation of chondrocytes and synthesis of ECM (Muller et al., 2020). Intraarticular injection of FGF18 attenuates the degradation of GAG, proteoglycan and COL2, inhibits the expression of MMP-13, reduces cartilage degeneration, down-regulates OARSI score, and ultimately promotes the regeneration and repairs of degenerated cartilage (Yao et al., 2019; Muller et al., 2020).

Sprifermin is a new truncated rhFGF18 which acts through the fibroblast growth factor receptor 3 (FGFR3). Sprifermin is roughly five times more potent in binding to this receptor compared to the natural FGF18 (Reker et al., 2020). Sprifermin exhibits strong capability in stimulating chondrocyte proliferation, stabilizing anabolic chondrocyte phenotype, promoting ECM synthesis, inhibiting proteolytic enzyme activity, increasing cartilage thickness, etc. (Hochberg et al., 2019; Muller et al., 2020; Reker et al., 2020). Sprifermin displays great potential in the treatment of OA and is expected to be marketed soon.

Sprifermin not only stimulates chondrocyte proliferation and chondrogenesis *in vitro*, but also effectively promotes cartilage repair *in vivo* (Ellsworth et al., 2002; Moore et al., 2005). Intraarticular administration of Sprifermin boosts chondrocyte proliferation, promotes cartilage anabolism, and improves cartilage biomechanical and histological properties in OA patients who are scheduled to undergo knee arthroplasty (Dahlberg et al., 2016). Through MRI observation, Roemer et al. confirmed that Sprifermin not only promotes chondrocyte proliferation in OA patients, but also plays an effective therapeutic role on bone marrow injury (Roemer et al., 2016). Animal experimental model has demonstrated that intraarticular injection of Sprifermin markedly prevents cartilage degradation and promotes the repair of damaged cartilage in traumatic OA model (Moore et al., 2005). In summary, clinical trials, *in vitro* experiments and animal experiments all indicate that Sprifermin possesses powerful performance in boosting chondrocyte proliferation and cartilage repair.

Sprifermin has exhibited outstanding prospects in promoting cartilage repair, and intraarticular application of Sprifermin is safe and reliable (Zeng et al., 2021). A comprehensive assessment of the specific role of Sprifermin in promoting cartilage repair and the evaluation of its potential molecular mechanisms are conducive to further improvement of Sprifermin.

## Mechanism of Action of Sprifermin

Sprifermin can activate FGFR3 on the surface of chondrocytes and drive chondrocyte proliferation and ECM synthesis (Davidson et al., 2005). In both monolayer and three-dimensional culture models of porcine chondrocytes, it was observed that Sprifermin not only increased the expression of chondrocyte markers, but also decreased COL1 expression level, increased the proportion of COL2:COL1, and maintained the chondrocyte phenotype (Gigout et al., 2017). In addition, Sprifermin does not increase the activity of proteolytic enzymes and expression of hypertrophy markers in three-dimensional culture, indicating that Sprifermin does not exacerbate catabolism and chondrocyte hypertrophy, and is able to safely and effectively maintain endochondral homeostasis (Kapoor et al., 2011; Reker et al., 2017).

**FIGURE 1:**
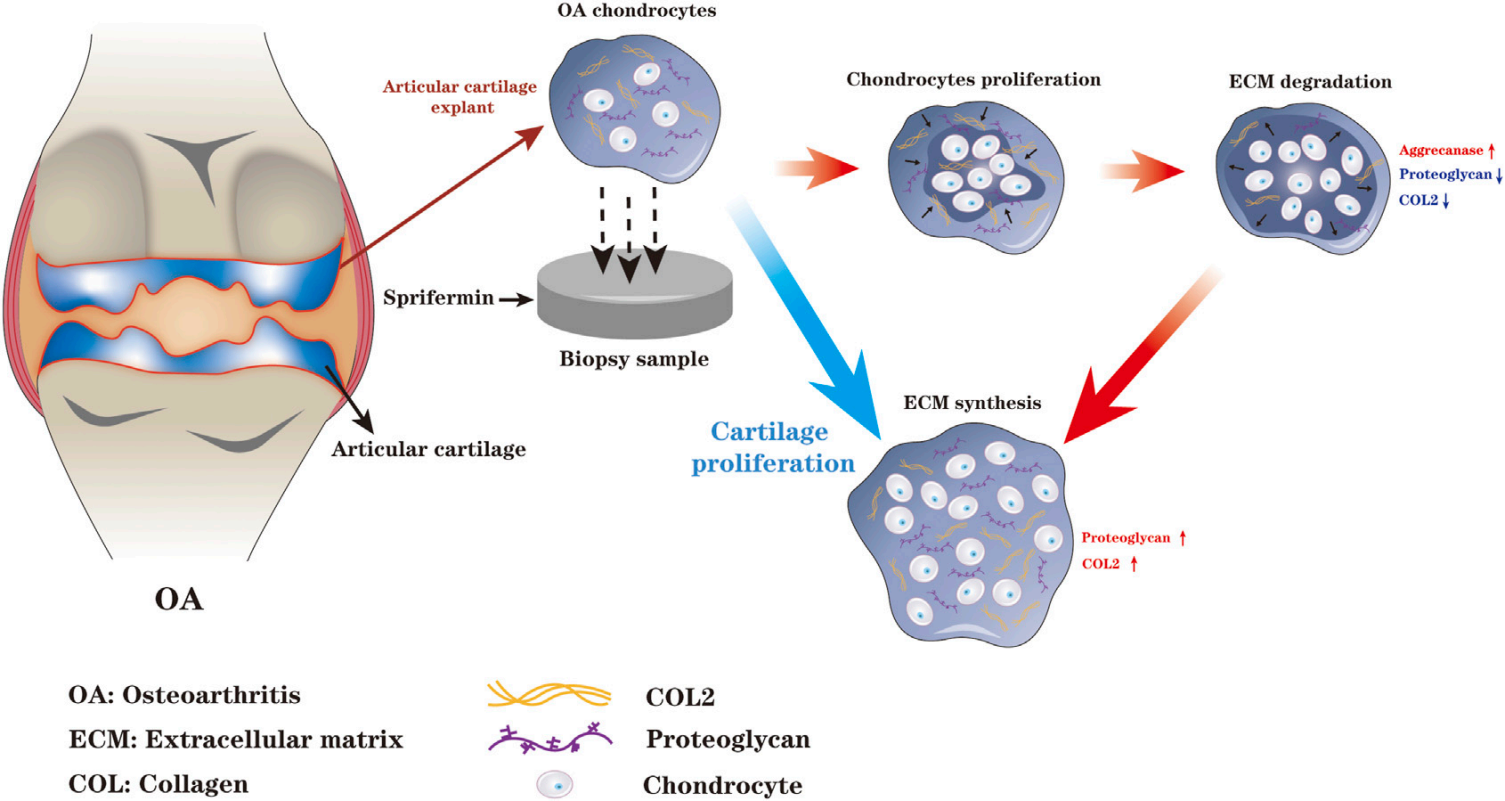
When Sprifermin acts on chondrocytes, there is a biphasic ECM remodeling process. The chondrocyte proliferation and ECM degradation occurs in the early-phase and ECM synthesis occurs in the late-phase. The biphasic ECM remodeling ensures that Sprifermin efficiently boosts chondrocyte proliferation, ECM synthesis, and ultimately promotes cartilage regeneration (The figure is made by adobe illustrator 2021).

The effect of Sprifermin on cartilage repair may be closely related to its ability to promote biphasic ECM remodeling process. Initially, Reker et al. reported that there may be a biphasic ECM remodeling process when Sprifermin acts on bovine articular cartilage explant, which is specifically manifested as the production of proteoglycan degradation enzyme is earlier than that of COL2 synthesis enzyme (Reker et al., 2017). In addition, chondrocytes monolayer and three-dimensional culture both displayed an inverse linear relationship between early chondrocyte proliferation and ECM synthesis, which further corroborated the above point of view (Gigout et al., 2017). In 2020, Reker and colleagues further elaborated the details of Sprifermin-induced biphasic process of ECM remodeling in human knee OA articular cartilage *ex vivo* (Reker et al., 2020). When Sprifermin promotes chondrogenesis, chondrocyte proliferation occurs in the early-phase and ECM synthesis occurs in the late-phase. The biphasic ECM remodeling process is firstly characterized by increased aggrecanase activity, leading to degradation of proteoglycan and COL2, followed by enlarged compartment and chondrocyte proliferation space, and thus synthesis of a growing amount of proteoglycan and COL2, while the metabolic activity is maintained. Perhaps the biphasic ECM remodeling is because of the expansion of chondrocyte population is limited by the surrounding ECM. When Sprifermin stimulates the resting chondrocytes in the ECM, the chondrocytes will induce ECM degradation to enlarge the compartment and allow chondrocytes to proliferate, which in turn produces more ECM and ultimately promotes chondrogenesis. Reker and colleagues speculated that matrix degradation is a prerequisite for the initiation of chondrocyte proliferation during ECM remodeling, the maintenance of the delicate balance between chondrocyte proliferation and ECM synthesis synergistically promotes cartilage regeneration (Reker et al., 2017; Reker et al., 2020) (Figure 1).

The retention time of Sprifermin in joint capsule is limited, and the therapeutic effect can be maximized by injection at reasonable time intervals. Studies have demonstrated that the application of Sprifermin for three consecutive weeks with repetition every 6 months exerts the best therapeutic effect on promoting cartilage proliferation (Hochberg et al., 2019; Sieber and Gigout, 2020). As for the long-term continuous medication of Sprifermin, although it promotes the chondrocytes proliferation to a certain extent, the acceleration of chondrogenic anabolism is not as good as intermittent repeated exposure. Similarly, it was reported that different exposure times of Sprifermin can lead to completely different or even opposite effect (Gigout et al., 2017). Long-term exposure to Sprifermin has a weaker down-regulation effect on COL1 than once a week exposure mode, discontinuation after initial exposure is necessary for maximizing Sprifermin’s anabolic potential (Gigout et al., 2017). For example, in the three-dimensional culture of porcine chondrocytes, after exposure to Sprifermin once a week for 4 weeks, the chondrocytes in the culture evidently increased and more GAG and hydroxyproline were synthesized (Sieber and Gigout, 2020); however, when the culture was continuously exposed to Sprifermin, the ECM synthesis was notably reduced (Rozenblatt-Rosen et al., 2002; Gigout et al., 2017). In conclusion, the intermittent exposure of Sprifermin activates the anabolic response instantly, which is beneficial to cartilage repair. However, permanent exposure might act on other signaling pathways and eventually exert a weaker role in promoting cartilage repair. This phenomenon may be attributed to desensitization (or negative feedback loop) of Sprifermin during long term exposure.

FGF18 acts via the FGFR in the cell membrane and regulates the runt-related transcription factor 2 (Runx2) *via* signaling molecules such as mitogen-activated protein kinase (MAPK) and phosphoinositide 3-kinase (PI3K), thereby regulating cartilage formation at the molecular level (Ellsworth et al., 2002; Jeon et al., 2012; Catheline et al., 2019). Gigout et al. confirmed that extracellular signal-regulated kinases 1 (ERK1) and ERK2 play an important role in Sprifermin signaling, such as promoting the formation of chondrocyte morphology and reducing the expression of COL1 (Gigout et al., 2017). FGF18 plays a crucial role in the development of bone and cartilage, as well as the dynamic balance of bone mineralization. Ohbayashi et al. documented that FGF18 has the highest affinity to FGFR3 and moderate affinity to FGFR2 (Ohbayashi et al., 2002). FGF18 directly regulates cartilage formation by acting on FGFR3. Besides, FGF18 regulates cartilage formation through other signaling pathways indirectly, for instance, FGF18 down-regulates the hedgehog signaling pathway activity in the interarticular region (Barnard et al., 2005; Clarke, 2020).

**FIGURE 2:**
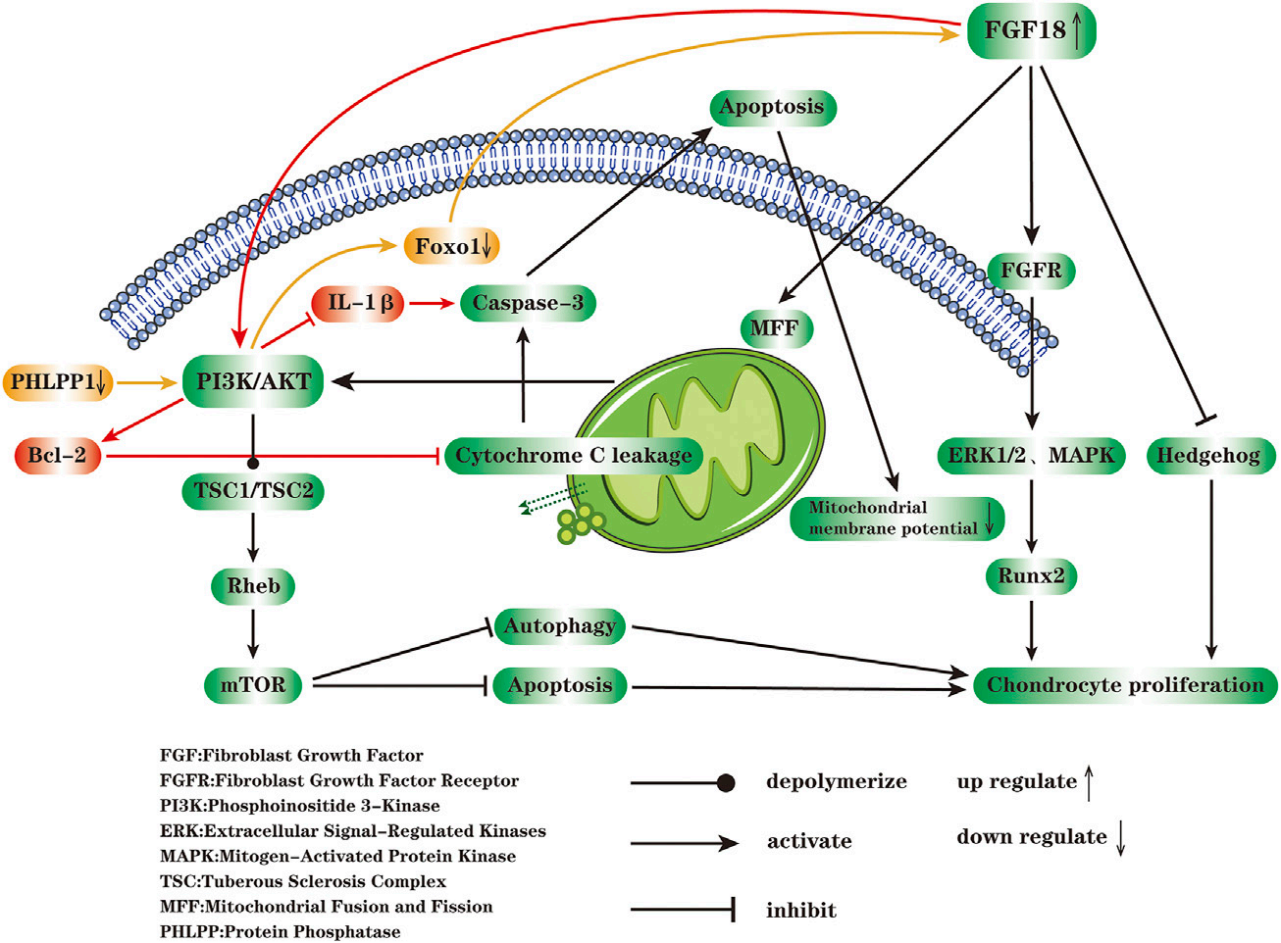
FGF18 acts on FGFR on the cell membrane and regulates chondrocyte proliferation through MAPK, ERK1/2 and hedgehog signaling pathways. The reduction of mitochondrial membrane potential and the leakage of cytochrome C into cytoplasm are closely related to cell apoptosis. FGF18 can maintain the balance of mitochondrial fusion and fission (MFF), restore the function and morphology of mitochondria, enhance the phosphorylation of PI3K and AKT, thereby depolymerizing TSC1/TSC2 and activating mTOR, ultimately inhibit apoptosis and autophagy of chondrocytes. After cytochrome C is released from mitochondria into the cytoplasm, it activates caspase-3 and causes apoptosis through a cascaded amplification reaction. FGF18 inhibits IL-1β-induced caspase3 activation through PI3K/AKT signaling pathway, enhances the expression of Bcl-2, and exerts an anti-apoptotic effect. Protein phosphatase1 (PHLPP1) is widely presented in chondrocytes during OA, and inhibits the proliferation of chondrocytes, differentiation, and matrix production. The decrease of PHLPP1 enhances the activity of Akt, and then down-regulates the transcription factor FoxO1 expression, followed by the increased expression of FGF18, and the enhanced phosphorylation of ERK1 or ERK2 through FGFR, and finally accelerates the proliferation of chondrocytes (The figure is made by adobe illustrator 2021).

At the cellular and molecular level, apoptosis is closely related to the occurrence and development of OA (Hwang and Kim, 2015). Mitochondria are organelles within the cytoplasm, bound by a double membrane. The membrane space and mitochondrial matrix jointly formed by outer membrane and inner membrane are capable of sensing and responding to external stressors. Generally speaking, mitochondria maintain cell homeostasis by generating energy and activating intracellular signaling pathways (Eisner et al., 2018). Reduced mitochondrial membrane potential and leakage of cytochrome C from mitochondria are landmark events of apoptosis. Under the stress environment, mitochondrial dysfunction may occur, which results in the inability to maintain enough protons to power oxidative phosphorylation (Shen et al., 2021). The PI3K/Akt pathway is a classical anti-apoptotic signal transduction pathway, which is regulated by a variety of upstream inflammatory mediators and could inhibit apoptosis and autophagy of chondrocytes (Xue et al., 2017). The activated Akt depolymerizes tuberous sclerosis complex 1 (TSC1)/TSC2 dimer, thereby abolishing the inhibitory effect on Rheb, and eventually activates mammalian target of rapamycin (mTOR) and downstream transcription factor eIF4E, regulates the expression of apoptosis-related genes (Xu et al., 2015). Phosphorylation of PI3K and Akt down-regulates the levels of various apoptotic factors such as Bax, Bim (Bcl-2-interacting mediator of cell death) and FoxO1 induced by IL-1β, thereby inhibiting cell apoptosis, restoring mitochondrial membrane potential and reducing the generation of ROS, finally promoting the proliferation and migration of chondrocytes. In most cases, the activity of PI3K/Akt signaling pathway is remarkedly blocked during the progression of OA (Wang et al., 2018; Zhang Y. et al., 2020). The balance of mitochondrial fusion and fission (MFF) can timely and effectively remove damaged mitochondria and restore the normal mechanism or function of mitochondria (Youle and van der Bliek, 2012). By maintaining MFF balance, FGF18 evidently restores the function and morphology of mitochondria, enhances the phosphorylation of PI3K and Akt, and exerts an efficient anti-osteoarthritis efficacy. Excessive activation of autophagy will exacerbate chondrocytes damage (Wang J. et al., 2020). The mTOR, an important downstream signaling molecule of PI3K/Akt, is involved in the negative regulation of autophagy (Li Y.-S. et al., 2016). After cytochrome C enters the cytoplasm, it will activate caspase-3 and induce apoptosis through cascading amplification reaction (Zhou et al., 2015). Bcl-2 is an important anti-apoptotic molecule, which can reduce the permeability of mitochondrial membrane to cytochrome C and inhibit the release of cytochrome C from mitochondria into the cytoplasm (Karaliotas et al., 2015). From the literature it is known that FGF18 activates the PI3/Akt signaling pathway to inhibit IL-1β-induced caspase-3 activation and Bcl-2 expression, thus playing an anti-apoptotic effect (Li Z. et al., 2016; Koundouros and Poulogiannis, 2018; Yao et al., 2019) (Figure 2).

## Cartilage Homeostasis

Cartilage is mainly composed of chondrocytes and gelatinous ECM. The natural ECM consists of COL2, COL1, GAG, etc. (Peng et al., 2021). The unique structure and composition of the cartilage ECM are important factors in maintaining cartilage homeostasis, providing a smooth joint articulation and enabling articular cartilage to withstand loads several times the body weight (Bailey et al., 2021; Peng et al., 2021). COL2 is the most abundant component in the ECM of cartilage, where it forms a fibrous network structure; while GAG absorbs a large amount of water to form gel, and thus maintaining the dilatability and elasticity of cartilage (Sherwood et al., 2014). Cartilage lacks blood vessels and nerves, and its self-repair ability is limited. Chondrocytes are the singular cell type of cartilage which maintain and regulate the osmotic pressure of cartilage. Death, abnormal activation and differentiation of chondrocytes, increased degradation of ECM, and excessive generation of proteolytic enzymes or inflammatory mediators can destruct cartilage homeostasis and induce or aggravate OA (Sandya et al., 2007; Muntyanu et al., 2016; Rim et al., 2020).

The imbalance of cartilage homeostasis is mainly the result of cartilage degeneration and inflammation (Ritter et al., 2013; Redondo et al., 2018; Xie et al., 2021). During OA, the initial stage of cartilage degeneration is manifested as superficial cartilage defect or fibrosis, followed by the formation of ulcers and cracks, and gradually expands to subchondral bone, making the cartilage thinner and thinner, leading to full-thickness cartilage defect (McCulloch et al., 2019; Chen et al., 2020). As the above events worsen, it can lead to subchondral bone exposure and intensified degeneration. Chondrodegeneration is an important event in the early stage of OA, and the articular inflammatory environment caused by degradation and destruction of cartilage in the middle and late stage ultimately lead to synovial hyperplasia and angiogenesis (Yu et al., 2016). Inflammatory factors produced in the pathological process directly act on chondrocytes and ECM, which can further destroy the cartilage homeostasis. Mechanical stress and acute injury up-regulate the gene expression of inflammatory factors IL-1, IL-6 and TNFα, thereby promoting the expression of ADAMTs, MMP-3, MMP-9 and MMP-13, leading to the degradation of COL2A1 and GAG (Yang et al., 2017). Proteolytic enzymes are involved in the synthesis, recombination, and repair of connective tissue. The external or internal injury, genetic abnormalities and irregular mechanical loading of cartilage could result in the imbalance of metabolic activity by overly enhancing the activity of proteolytic enzymes, and ultimately accelerate cartilage degradation (Choi et al., 2019). IL-1 has a strong ability to induce aggrecanolysis and up-regulate the synthesis of chondrodegrading enzymes, such as MMP-3, ADAMTS-4, and ADAMTS-5 (Kapoor et al., 2011; Na et al., 2020). IL-6 can efficiently reduce proteoglycan synthesis in normal cartilage *in vitro* (Wang W. et al., 2020). TNFα induces the synthesis of MMPs and other proteases in chondrocytes, meanwhile increases prostaglandin E2 (PGE2) level by stimulating the synthesis of cyclooxygenase-2, microsomal PGE synthase-1 and soluble phospholipase A2 (Fitzgerald et al., 2004). The cartilage degeneration and inflammatory microenvironment of cartilage can activate each other and jointly aggravate the destruction of cartilage homeostasis.

## Research Progress of Sprifermin in the Treatment of Cartilage Related Diseases

**FIGURE 3:**
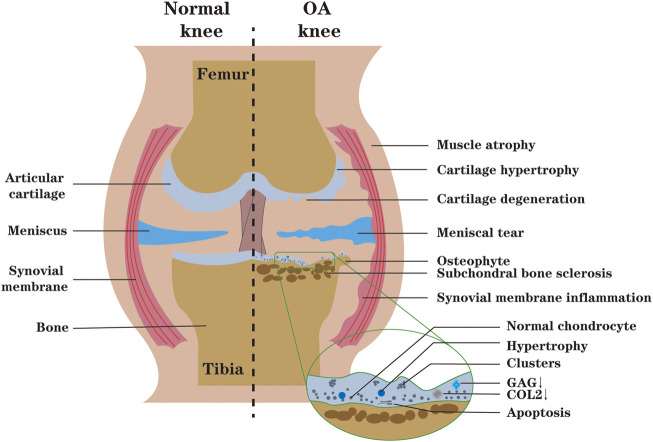
During OA, the synthesis of ECM (GAG, COL2) decreases, and cartilage degeneration occurs. Microscopically, the spatial arrangement of chondrocytes is changed from single to double strings, then to clusters. With the development of OA, chondrocytes undergo abnormal activation and even death, which intensifies the degradation and destruction of cartilage. The inflammatory environment of the joint may lead to synovial proliferation, biochemical and biomechanical changes in the articular cartilage, compensatory osteophyte formation, muscle atrophy and meniscus tears of the knee (The figure is made by adobe illustrator 2021).

OA is a heterogeneous disease involving the whole joint, and cartilage degeneration is the main feature (Shah and Mithoefer, 2020a). Under the microscope, articular cartilage damage can be assessed by analysis of tissue sections. OA cartilage can be characterized by the appearance of clusters of chondrocytes near the superficial layer, chondrocyte apoptosis in the deep and calcified layer, and disruption of ECM due to the degradation of collagen and proteoglycan. Although the proliferative activity of chondrocytes is also activated, the catabolic activity is much higher than the proliferative activity (Sandell and Aigner, 2001; Carlo and Loeser, 2008). As the disease progresses, the degrading enzymes produced by articular chondrocytes gradually increase, further aggravating the destruction of articular biomechanics and biochemistry (Dreier, 2010) (Figure 3).

A first-in-human study showed that intraarticular injection of Sprifermin efficiently promotes chondrocytes proliferation and has a positive effect on histological and biomechanical cartilage properties (Dahlberg et al., 2016). Quantitative measurement and ultrastructural analysis indicated that treatment with Sprifermin increases the synthesis of GAG and COL2 and promotes the formation of ECM connections across the cartilage, which helps damaged cartilage to complete the repair process (Gigout et al., 2017). In the *in vitro* repair model, mechanical and biochemical analysis displayed that the adhesion strength between cartilage surfaces is stronger and the contact area between core cartilage and cartilage rings is larger in the Sprifermin treatment group, indicating that Sprifermin effectively promotes the healing of articular cartilage defects and the integration between lateral cartilage (Sennett et al., 2018). In a sheep model, Power and colleagues compared the efficacy between Microfracture (MFX) and MFX with rhFGF18 in the treatment of articular cartilage defects, and they observed significant statistical differences in International Cartilage Repair Society (ICRS) tissue repair scores, tissue fill scores, and improved O'Driscoll scores at 6 months between the two groups. MFX combined with rhFGF18 promotes the formation of hyaline chondroid tissue compared to the MFX group (Power et al., 2014). Sennett et al. also reported that, compared with the control group, the Sprifermin treatment group has stronger cartilage-to-cartilage interface adhesion strength, more COL2 content, and larger contact area between core cartilage and annular cartilage, which indicates that Sprifermin can effectively promote the repair of damaged cartilage (Sennett et al., 2018).

The current literature shows that, compared with placebo treatment, administration of Sprifermin not only increases cartilage thickness in some locations of the joint (adds cartilage volume and thickness globally, or at some regions where cartilage thickness is expected to be static), but also notably reduces cartilage loss (Lohmander et al., 2014; Eckstein et al., 2015). Similarly, following Sprifermin treatment, the percentage of cartilage thickness of medial central tibia, lateral central tibia and medial central femoral condylar has the largest changes, indicating a strong sensitivity to Sprifermin, and the most obvious difference between Sprifermin and placebo group is in medial central tibia (Hochberg et al., 2019). This indicates that the high bearing area of the joint and the area where cartilage is prone to damage are more sensitive to Sprifermin (Hochberg et al., 2018; Eckstein et al., 2020). Roemer et al. confirmed that Sprifermin exerts a positive effect on cartilage morphology and improvement of bone marrow-induced lesions (BML), and no negative effects associated with Sprifermin are reported in other joint tissues (Roemer et al., 2016).

Sprifermin can specifically target FGFR3 of chondrocytes. Since FGFR3 is expressed in meniscus, Sprifermin is expected to achieve a therapeutical effect when meniscus is injured (Roemer et al., 2020). Studies by Lohmander et al. showed that after intra-articular injection of Sprifermin, cartilage thickness significantly increases in a dose-dependent manner (Lohmander et al., 2014). Gigout and colleagues confirmed that in monolayer culture of porcine chondrocytes and 3D culture of human and porcine chondrocytes, Sprifermin could promote chondrocyte proliferation dose-dependently (Gigout et al., 2017). (Lohmander et al., 2014). Intra-articular injection of FGF18 can activate FGFR3C, alleviate the cartilage degradation of rat post-traumatic osteoarthritis (PTOA), increase the deposition of COL2, and inhibit the expression of MMP-13 (Karuppaiah et al., 2016; Onuora, 2021). When OA occurs, the expression of FGFR3 in chondrocytes is down-regulated, usually accompanied by increased FGFR1 level, while FGF18 can up-regulate the expression of FGFR3 and down-regulate FGFR1 level in chondrocytes (Yao et al., 2019). In addition, FGF18 expression and FGFR3 activation in growth plate chondrocytes inhibit proliferation and hypertrophy of chondrocytes (Karuppaiah et al., 2016). Ohbayashi et al. reported that FGF18 is required for both osteogenesis and chondrogenesis in bone development, it promotes both proliferation of osteogenic mesenchymal cells and terminal differentiation to mature osteoblasts (Ohbayashi et al., 2002).

There are many studies which focused on the anti-OA effect of FGF18 *in vivo*. Power et al. found that, through animal experiments, microfracture surgery combined with intraarticular injection of rhFGF18 effectively can promote the repair of articular cartilage defects of the medial femoral condyle (Power et al., 2014). Moore et al. also demonstrated that intraarticular injection of FGF18 stimulates cartilage repair in a rat meniscus tear model, giving rise to a dose-dependent increase in cartilage thickness and a significant reduction in cartilage degeneration scores (Moore et al., 2005). Similarly, in another OA rat model, administration of rhFGF18 could prevent cartilage degeneration (Mori et al., 2014). RhFGF18 is expected to protect articular cartilage from injury. In a five-year phase II clinical trial, Eckstein et al. have proved that intra-articular injection of Sprifermin can promote knee cartilage regeneration in OA patients. Sprifermin is the first DMOAD candidate drug that can promote the regeneration of damaged articular cartilage (Hochberg et al., 2019; Eckstein et al., 2020; Eckstein et al., 2021). Initially, Hochberg et al. reported that intra-articular injection of Sprifermin efficiently increases the cartilage thickness of femorotibial joint, compared with other doses and frequencies, the intra-articular administration of 100 μg every 6 months is most effective (Hochberg et al., 2019). Through a large sample study, they further confirmed that Sprifermin increases the thickness of the cartilage in all parts of the femorotibial joint in OA patients, and the effect is more obvious in high load-bearing areas such as the central medial tibia (Eckstein et al., 2020). Based on above, Eckstein et al. further evaluated the effectiveness and safety of Sprifermin in patients with OA. Following treatment with Sprifermin, the WOMAC pain score improved by approximately 50% in all groups with different doses and frequencies. Sprifermin treatment can maintain long-term structural modification of articular cartilage, hereby they confirmed that Sprifermin can not only modify the structure of articular cartilage, but also effectively alleviate clinical symptoms (Eckstein et al., 2021).

The promotion effect of Sprifermin on chondrogenesis depends on the inflammatory environment to some extent. Under inflammatory state, the regulative effect of Sprifermin on articular cartilage anabolism is obviously attenuated (Reker et al., 2017). For instance, when explants are co-cultured with pro-inflammatory cytokines prior to Sprifermin, almost no changes in COL2 synthesis and ECM degradation markers are observed following Sprifermin treatment. Other studies have confirmed that continuous co-culture of oncostatin M + TNFα almost eliminates Sprifermin-stimulated COL2 synthesis during the whole culture period (Reker et al., 2017). In clinical work, matching the appropriate treatment method with the corresponding patient is very important for the effective treatment of OA, due to that the cartilage regeneration effect of Sprifermin may be largely affected by the inflammation of the joint cavity. In view of the above studies, Sprifermin may be a more effective DMOAD for OA patients with mild synovitis or low levels of pro-inflammatory factors. Sprifermin injection in the knee cavity in patients with early OA (Kellgren-Lawrence grade 1 or 2) combined with other oral agents can delay or even reverse the progression of OA. For severe OA patients (Kellgren-Lawrence grade 3 or 4), Sprifermin is expected to delay the time to total knee replacement (Table 3).

**TABLE 3:** Table of the transition from animal experiment to clinical trial.

Research conclusion	Research type	References
The proliferation of chondrocytes and production of ECM increased after addition of FGF18 into the medium of primary articular chondrocytes. FGF18 promotes the expression of FGFR3 and FGFR2 in chondrocytes. They hypothesize that FGF18 acts as a trophic factor for chondrocytes and plays an autocrine role in the biology of normal articular cartilage	*In vitro* Experiment	Ellsworth et al. (2002)
FGF18 increases cartilage thickness of the tibial plateau in a dose-dependent manner, and high doses of FGF18 can promote the remodeling of subchondral bone. In an *in vivo* model of OA, research confirms FGF18 is the first anabolic agent, which helps to facilitate cartilage repair	Animal Experiment	Moore et al. (2005)
Animal experiments show that intra-articular infiltration with rhFGF-18 improves cartilage repair with a phenotype similar to hyaline cartilage. No adverse events are encountered throughout the study, which indicates that rhFGF-18 is safe and efficacious, therefore it can be used as an excellent early treatment choice for OA	Animal Experiment	Power et al. (2014)
The dose-dependent effect of Sprifermin on the cartilage thickness across the total femorotibial joint and in the LFTC (lateral femorotibial cartilage) are consistent, there are no local or systemic safety concerns and complications	Clinic Trial	Lohmander et al. (2014)
The clinic trial evaluates the effects of intra-articular injection of Sprifermin in different doses and frequencies on articular cartilage structure in patients with OA. Results show that intra-articular injection of Sprifermin at 100 μg every 6 months exerts the most improvement in cartilage thickness after 2 years. However, the study has not yet determined the durability of response	Clinic Trial	Hochberg et al. (2019)
In addition to the positive effect of Sprifermin on cartilage thickness, the post-hoc analysis shows that Sprifermin also has a dose-dependent positive effect on cartilage morphology and bone marrow lesions in the patellofemoral joint, and Sprifermin treatment has no deleterious effects on other joint tissues	Clinic Trial	Roemer et al. (2020)
The clinic trial shows that intraarticular injection of Sprifermin can increase the thickness of the femorotibial joint cartilage, which is most obvious for the areas with high load-bearing and pre-existing cartilage damage. Sprifermin is safe and effective in OA patients, it can improve WOMAC pain scores. Sprifermin not only changes the structure of articular cartilage, but also effectively relieves clinical symptoms	Clinic Trial	Eckstein et al. (2021)

## Limitation

At present, most of the experiments on Sprifermin are still in the animal stage, and the application of Sprifermin in human body is rare. The effect of Sprifermin may be different between human and animal cartilage. Although Sprifermin can promote cartilage regeneration and increase cartilage thickness, it remains unclear whether cartilage can regenerate according to the natural cartilage structure. Additionally, the dose and method of administration of Sprifermin are still controversial. Due to the limited volume of articular cavity, there is no consensus on how to achieve the maximum therapeutic effect with the minimum dose of Sprifermin. For patients with symptomatic knee OA, the follow up time after intraarticular injection of Sprifermin is yet not long enough, and its clinical effectiveness remains uncertain. The long-term effect of Sprifermin in clinical patients requires longer follow-up time and more clinical samples, and more clinical trials are needed to prove that articular cartilage structural modification in OA patients can be translated into symptomatic benefit.

## Conclusion

Thus far, there is no satisfactory drug or method for the treatment of OA. Sprifermin is a promising drug of DMOAD, displaying unique advantage in maintaining cartilage homeostasis. Sprifermin can effectively promote chondrocyte proliferation and ECM synthesis, maintain chondrocyte phenotype. Following Sprifermin treatment, the enlarged chondrocyte population produce transparent ECM, and there is a biphasic ECM remodeling process, which remarkably amplifies the efficiency of cartilage regeneration. The functions of Sprifermin might be closely related to its anti-inflammatory and maintenance of mitochondrial performance. Clinical trials have confirmed that intermittent injections of Sprifermin in the knee joint once a week can maximize the effect of anti-OA. Therefore, Sprifermin is expected to become a safe and effective drug for delaying or even reversing cartilage damage-related diseases. Although some progress has been made, the specific mechanism of its action is still unclear. Before Sprifermin can be used on a large scale in clinical treatment of cartilage injury-related diseases, its long-term safety and effectiveness still need to be evaluated by further studies.

## References

[B1] Alexander L. A. M. Ln D. Eg Z. Is D. Ay K. Ss R. (2020). Pharmacological Management of Osteoarthritis with a Focus on Symptomatic Slow-Acting Drugs. J. Clin. Rheumatol. 27, e533–e539. 10.1097/rhu.0000000000001507 32732520

[B2] Antunes B. P. Vainieri M. L. Alini M. Monsonego-Ornan E. Grad S. Yayon A. (2020). Enhanced Chondrogenic Phenotype of Primary Bovine Articular Chondrocytes in Fibrin-Hyaluronan Hydrogel by Multi-Axial Mechanical Loading and FGF18. Acta Biomater. 105, 170–179. 10.1016/j.actbio.2020.01.032 31982592

[B3] Bailey K. N. Nguyen J. Yee C. S. Dole N. S. Dang A. Alliston T. (2021). Mechanosensitive Control of Articular Cartilage and Subchondral Bone Homeostasis in Mice Requires Osteocytic Transforming Growth Factor β Signaling. Arthritis Rheumatol. 73, 414–425. 10.1002/art.41548 33022131 PMC9026749

[B4] Barnard J. C. Williams A. J. Rabier B. Chassande O. Samarut J. Cheng S.-y. (2005). Thyroid Hormones Regulate Fibroblast Growth Factor Receptor Signaling during Chondrogenesis. Endocrinology 146, 5568–5580. 10.1210/en.2005-0762 16150908

[B5] Boylan M. Anderson M. J. Ornitz D. M. Lewandoski M. (2020). The Fgf8 Subfamily (Fgf8, Fgf17 and Fgf18) Is Required for Closure of the Embryonic Ventral Body wall. Development 147, dev189506. 10.1242/dev.189506 32907848 PMC7595690

[B6] Brody L. T. (2015). Knee Osteoarthritis: Clinical Connections to Articular Cartilage Structure and Function. Phys. Ther. Sport 16, 301–316. 10.1016/j.ptsp.2014.12.001 25783021

[B7] Bükülmez H. Khan F. Bartels C. F. Murakami S. Ortiz‐Lopez A. Sattar A. (2014). Protective Effects of C‐Type Natriuretic Peptide on Linear Growth and Articular Cartilage Integrity in a Mouse Model of Inflammatory Arthritis. Arthritis Rheumatol. 66, 78–89. 10.1002/art.38199 24449577 PMC4034591

[B8] Carlo M. D. Loeser R. F. (2008). Cell Death in Osteoarthritis. Curr. Rheumatol. Rep. 10, 37–42. 10.1007/s11926-008-0007-8 18457610

[B9] Catheline S. E. Hoak D. Chang M. Ketz J. P. Hilton M. J. Zuscik M. J. (2019). Chondrocyte‐Specific RUNX2 Overexpression Accelerates Post‐traumatic Osteoarthritis Progression in Adult Mice. J. Bone Miner Res. 34, 1676–1689. 10.1002/jbmr.3737 31189030 PMC7047611

[B10] Chen L. Zheng J. J. Y. Li G. Yuan J. Ebert J. R. Li H. (2020). Pathogenesis and Clinical Management of Obesity-Related Knee Osteoarthritis: Impact of Mechanical Loading. J. Orthopaedic Translation 24, 66–75. 10.1016/j.jot.2020.05.001 PMC7349942 32695606

[B11] Choi M. Jo J. Park J. Kang H. K. Park Y. (2019). NF-B Signaling Pathways in Osteoarthritic Cartilage Destruction. Cells 8, 734. 10.3390/cells8070734 PMC6678954 31319599

[B12] Clarke J. (2020). Opioid and Hedgehog Signalling Pathways Converge to Modulate OA. Nat. Rev. Rheumatol. 16, 297. 10.1038/s41584-020-0429-x 32358543

[B13] Dahlberg L. E. Aydemir A. Muurahainen N. Gühring H. Fredberg Edebo H. Krarup-Jensen N. (2016). A First-In-Human, Double-Blind, Randomised, Placebo-Controlled, Dose Ascending Study of Intra-articular rhFGF18 (Sprifermin) in Patients with Advanced Knee Osteoarthritis. Clin. Exp. Rheumatol. 34, 445–450. 27050139

[B14] Davidson D. Blanc A. Filion D. Wang H. Plut P. Pfeffer G. (2005). Fibroblast Growth Factor (FGF) 18 Signals through FGF Receptor 3 to Promote Chondrogenesis. J. Biol. Chem. 280, 20509–20515. 10.1074/jbc.M410148200 15781473

[B15] Davies L. C. Blain E. J. Gilbert S. J. Caterson B. Duance V. C. (2008). The Potential of IGF-1 and TGFβ1 for Promoting "Adult" Articular Cartilage Repair: AnIn VitroStudy. Tissue Eng. A 14, 1251–1261. 10.1089/ten.tea.2007.0211 18399732

[B16] Deng M. Hu Y. Zhang Z. Zhang H. Qu Y. Shao G. (2021). Unicondylar Knee Replacement versus Total Knee Replacement for the Treatment of Medial Knee Osteoarthritis: a Systematic Review and Meta-Analysis. Arch. Orthop. Trauma Surg. 141, 1361–1372. 10.1007/s00402-021-03790-7 33512583 PMC8295078

[B17] Dreier R. (2010). Hypertrophic Differentiation of Chondrocytes in Osteoarthritis: the Developmental Aspect of Degenerative Joint Disorders. Arthritis Res. Ther. 12, 216. 10.1186/ar3117 20959023 PMC2990991

[B18] Eckstein F. Hochberg M. C. Guehring H. Moreau F. Ona V. Bihlet A. R. (2021). Long-term Structural and Symptomatic Effects of Intra-articular Sprifermin in Patients with Knee Osteoarthritis: 5-year Results from the FORWARD Study. Ann. Rheum. Dis. 80, 1062–1069. 10.1136/annrheumdis-2020-219181 PMC8292562 33962962

[B19] Eckstein F. Kraines J. L. Aydemir A. Wirth W. Maschek S. Hochberg M. C. (2020). Intra-articular Sprifermin Reduces Cartilage Loss in Addition to Increasing Cartilage Gain Independent of Location in the Femorotibial Joint: post-hoc Analysis of a Randomised, Placebo-Controlled Phase II Clinical Trial. Ann. Rheum. Dis. 79, 525–528. 10.1136/annrheumdis-2019-216453 32098758 PMC7147175

[B20] Eckstein F. Wirth W. Guermazi A. Maschek S. Aydemir A. (2015). Brief Report: Intraarticular Sprifermin Not Only Increases Cartilage Thickness, but Also Reduces Cartilage Loss: Location‐Independent Post Hoc Analysis Using Magnetic Resonance Imaging. Arthritis Rheumatol. 67, 2916–2922. 10.1002/art.39265 26138203 PMC5061102

[B21] Eisner V. Picard M. Hajnóczky G. (2018). Mitochondrial Dynamics in Adaptive and Maladaptive Cellular Stress Responses. Nat. Cel Biol 20, 755–765. 10.1038/s41556-018-0133-0 PMC6716149 29950571

[B22] Ellsworth J. L. Berry J. Bukowski T. Claus J. Feldhaus A. Holderman S. (2002). Fibroblast Growth Factor-18 Is a Trophic Factor for Mature Chondrocytes and Their Progenitors. Osteoarthritis and Cartilage 10, 308–320. 10.1053/joca.2002.0514 11950254

[B23] Ernstbrunner L. Imam M. A. Andronic O. Perz T. Wieser K. Fucentese S. F. (2018). Lateral Unicompartmental Knee Replacement: a Systematic Review of Reasons for Failure. Int. Orthopaedics (Sicot) 42, 1827–1833. 10.1007/s00264-017-3662-4 29030653

[B24] Fitzgerald J. B. Jin M. Dean D. Wood D. J. Zheng M. H. Grodzinsky A. J. (2004). Mechanical Compression of Cartilage Explants Induces Multiple Time-dependent Gene Expression Patterns and Involves Intracellular Calcium and Cyclic AMP. J. Biol. Chem. 279, 19502–19511. 10.1074/jbc.M400437200 14960571

[B25] Gigout A. Guehring H. Froemel D. Meurer A. Ladel C. Reker D. (2017). Sprifermin (rhFGF18) Enables Proliferation of Chondrocytes Producing a Hyaline Cartilage Matrix. Osteoarthritis and Cartilage 25, 1858–1867. 10.1016/j.joca.2017.08.004 28823647

[B26] Harasymowicz N. S. Choi Y. R. Wu C. L. Iannucci L. Tang R. Guilak F. (2020). Intergenerational Transmission of Diet‐Induced Obesity, Metabolic Imbalance, and Osteoarthritis in Mice. Arthritis Rheumatol. 72, 632–644. 10.1002/art.41147 31646754 PMC7113102

[B27] Hochberg M. C. Altman R. D. April K. T. Benkhalti M. Guyatt G. McGowan J. (2012). American College of Rheumatology 2012 Recommendations for the Use of Nonpharmacologic and Pharmacologic Therapies in Osteoarthritis of the Hand, Hip, and Knee. Arthritis Care Res. 64, 465–474. 10.1002/acr.21596 22563589

[B28] Hochberg M. C. Guermazi A. Guehring H. Aydemir A. Wax S. Fleuranceau-Morel P. (2019). Effect of Intra-articular Sprifermin vs Placebo on Femorotibial Joint Cartilage Thickness in Patients with Osteoarthritis. JAMA 322, 1360–1370. 10.1001/jama.2019.14735 31593273 PMC6784851

[B29] Hochberg M. Guermazi A. Guehring H. Aydemir A. Wax S. Fleuranceau-Morel P. (2018). Efficacy and Safety of Intra-articular Sprifermin in Symptomatic Radiographic Knee Osteoarthritis: Pre-specified Analysis of 3-year Data from a 5-year Randomized, Placebo-Controlled, Phase II Study. Osteoarthritis and Cartilage 26, S26–S27. 10.1016/j.joca.2018.02.069

[B30] Hu M. C.-T. Qiu W. R. Wang Y.-p. Hill D. Ring B. D. Scully S. (1998). FGF-18, a Novel Member of the Fibroblast Growth Factor Family, Stimulates Hepatic and Intestinal Proliferation. Mol. Cel Biol 18, 6063–6074. 10.1128/mcb.18.10.6063 PMC109192 9742123

[B31] Hung I. H. Schoenwolf G. C. Lewandoski M. Ornitz D. M. (2016). A Combined Series of Fgf9 and Fgf18 Mutant Alleles Identifies Unique and Redundant Roles in Skeletal Development. Develop. Biol. 411, 72–84. 10.1016/j.ydbio.2016.01.008 26794256 PMC4801039

[B32] Hunter D. J. Pike M. C. Jonas B. L. Kissin E. Krop J. McAlindon T. (2010). Phase 1 Safety and Tolerability Study of BMP-7 in Symptomatic Knee Osteoarthritis. BMC Musculoskelet. Disord. 11, 232. 10.1186/1471-2474-11-232 20932341 PMC2958989

[B33] Hwang H. Kim H. (2015). Chondrocyte Apoptosis in the Pathogenesis of Osteoarthritis. Ijms 16, 26035–26054. 10.3390/ijms161125943 26528972 PMC4661802

[B34] Jeon E. Yun Y.-R. Kang W. Lee S. Koh Y.-H. Kim H.-W. (2012). Investigating the Role of FGF18 in the Cultivation and Osteogenic Differentiation of Mesenchymal Stem Cells. PLoS One 7, e43982. 10.1371/journal.pone.0043982 22937141 PMC3427245

[B35] Kapoor M. Martel-Pelletier J. Lajeunesse D. Pelletier J.-P. Fahmi H. (2011). Role of Proinflammatory Cytokines in the Pathophysiology of Osteoarthritis. Nat. Rev. Rheumatol. 7, 33–42. 10.1038/nrrheum.2010.196 21119608

[B36] Karaliotas G. I. Mavridis K. Scorilas A. Babis G. C. (2015). Quantitative Analysis of the mRNA Expression Levels of BCL2 and BAX Genes in Human Osteoarthritis and normal Articular Cartilage: An Investigation into Their Differential Expression. Mol. Med. Rep. 12, 4514–4521. 10.3892/mmr.2015.3939 26081683

[B37] Karsdal M. A. Michaelis M. Ladel C. Siebuhr A. S. Bihlet A. R. Andersen J. R. (2016). Disease-modifying Treatments for Osteoarthritis (DMOADs) of the Knee and Hip: Lessons Learned from Failures and Opportunities for the Future. Osteoarthritis and Cartilage 24, 2013–2021. 10.1016/j.joca.2016.07.017 27492463

[B38] Karuppaiah K. Yu K. Lim J. Chen J. Smith C. Long F. (2016). FGF Signaling in the Osteoprogenitor Lineage Non-autonomously Regulates Postnatal Chondrocyte Proliferation and Skeletal Growth. Development 143, 1811–1822. 10.1242/dev.131722 27052727 PMC4874483

[B39] Katz J. N. Arant K. R. Loeser R. F. (2021). Diagnosis and Treatment of Hip and Knee Osteoarthritis. JAMA 325, 568–578. 10.1001/jama.2020.22171 33560326 PMC8225295

[B40] Khosravi F. Ahmadvand N. Bellusci S. Sauer H. (2021). The Multifunctional Contribution of FGF Signaling to Cardiac Development, Homeostasis, Disease and Repair. Front. Cel Dev. Biol. 9, 672935. 10.3389/fcell.2021.672935 PMC8169986 34095143

[B41] Koundouros N. Poulogiannis G. (2018). Phosphoinositide 3-Kinase/Akt Signaling and Redox Metabolism in Cancer. Front. Oncol. 8, 160. 10.3389/fonc.2018.00160 29868481 PMC5968394

[B42] Krishnan Y. Grodzinsky A. J. (2018). Cartilage Diseases. Matrix Biol. 71-72, 51–69. 10.1016/j.matbio.2018.05.005 29803938 PMC6146013

[B43] Li J. Wang X. Ruan G. Zhu Z. Ding C. (2021). Sprifermin: a Recombinant Human Fibroblast Growth Factor 18 for the Treatment of Knee Osteoarthritis. Expert Opin. Investig. Drugs 30, 923–930. 10.1080/13543784.2021.1972970 34427483

[B44] Li Y.-S. Zhang F.-J. Zeng C. Luo W. Xiao W.-F. Gao S.-G. (2016a). Autophagy in Osteoarthritis. Jt. Bone Spine 83, 143–148. 10.1016/j.jbspin.2015.06.009 26453105

[B45] Li Z. Zhu T. Fan W. (2016b). Osteochondral Autograft Transplantation or Autologous Chondrocyte Implantation for Large Cartilage Defects of the Knee: a Meta-Analysis. Cell Tissue Bank 17, 59–67. 10.1007/s10561-015-9515-8 26068598

[B46] Liu G. Chen T. Ding Z. Wang Y. Wei Y. Wei X. (2021). Inhibition of FGF‐FGFR and VEGF‐VEGFR Signalling in Cancer Treatment. Cell Prolif 54, e13009. 10.1111/cpr.13009 33655556 PMC8016646

[B47] Lohmander L. S. Hellot S. Dreher D. Krantz E. F. W. Kruger D. S. Guermazi A. (2014). Intraarticular Sprifermin (Recombinant Human Fibroblast Growth Factor 18) in Knee Osteoarthritis: a Randomized, Double-Blind, Placebo-Controlled Trial. Arthritis Rheumatol. 66, 1820–1831. 10.1002/art.38614 24740822

[B48] Lv X. Sun C. Hu B. Chen S. Wang Z. Wu Q. (2020). Simultaneous Recruitment of Stem Cells and Chondrocytes Induced by a Functionalized Self-Assembling Peptide Hydrogel Improves Endogenous Cartilage Regeneration. Front. Cel Dev. Biol. 8, 864. 10.3389/fcell.2020.00864 PMC7493663 33015049

[B49] Malemud C. J. (2019). Inhibition of MMPs and ADAM/ADAMTS. Biochem. Pharmacol. 165, 33–40. 10.1016/j.bcp.2019.02.033 30826330 PMC6557692

[B50] Martínez-Moreno D. Jiménez G. Gálvez-Martín P. Rus G. Marchal J. A. (2019). Cartilage Biomechanics: A Key Factor for Osteoarthritis Regenerative Medicine. Biochim. Biophys. Acta (Bba) - Mol. Basis Dis. 1865, 1067–1075. 10.1016/j.bbadis.2019.03.011 30910703

[B51] Maruoka Y. Ohbayashi N. Hoshikawa M. Itoh N. Hogan B. L. M. Furuta Y. (1998). Comparison of the Expression of Three Highly Related Genes, Fgf8, Fgf17 and Fgf18, in the Mouse Embryo. Mech. Develop. 74, 175–177. 10.1016/s0925-4773(98)00061-6 9651520

[B52] McCulloch K. Huesa C. Dunning L. Litherland G. J. Van ‘T Hof R. J. Lockhart J. C. (2019). Accelerated post Traumatic Osteoarthritis in a Dual Injury Murine Model. Osteoarthritis and Cartilage 27, 1800–1810. 10.1016/j.joca.2019.05.027 31283983

[B53] Mehana E.-S. E. Khafaga A. F. El-Blehi S. S. (2019). The Role of Matrix Metalloproteinases in Osteoarthritis Pathogenesis: An Updated Review. Life Sci. 234, 116786. 10.1016/j.lfs.2019.116786 31445934

[B54] Moore E. E. Bendele A. M. Thompson D. L. Littau A. Waggie K. S. Reardon B. (2005). Fibroblast Growth Factor-18 Stimulates Chondrogenesis and Cartilage Repair in a Rat Model of Injury-Induced Osteoarthritis. Osteoarthritis and Cartilage 13, 623–631. 10.1016/j.joca.2005.03.003 15896984

[B55] Mori Y. Saito T. Chang S. H. Kobayashi H. Ladel C. H. Guehring H. (2014). Identification of Fibroblast Growth Factor-18 as a Molecule to Protect Adult Articular Cartilage by Gene Expression Profiling. J. Biol. Chem. 289, 10192–10200. 10.1074/jbc.M113.524090 24577103 PMC3974988

[B56] Müller S. Lindemann S. Gigout A. (2020). Effects of Sprifermin, IGF1, IGF2, BMP7, or CNP on Bovine Chondrocytes in Monolayer and 3D Culture. J. Orthop. Res. 38, 653–662. 10.1002/jor.24491 31608492 PMC7065224

[B57] Muntyanu A. Abji F. Liang K. Pollock R. A. Chandran V. Gladman D. D. (2016). Differential Gene and Protein Expression of Chemokines and Cytokines in Synovial Fluid of Patients with Arthritis. Arthritis Res. Ther. 18, 296. 10.1186/s13075-016-1196-6 27964744 PMC5154157

[B58] Na H. S. Park J.-S. Cho K.-H. Kwon J. Y. Choi J. Jhun J. (2020). Interleukin-1-Interleukin-17 Signaling Axis Induces Cartilage Destruction and Promotes Experimental Osteoarthritis. Front. Immunol. 11, 730. 10.3389/fimmu.2020.00730 32431699 PMC7214841

[B59] Ohbayashi N. Shibayama M. Kurotaki Y. Imanishi M. Fujimori T. Itoh N. (2002). FGF18 Is Required for normal Cell Proliferation and Differentiation during Osteogenesis and Chondrogenesis. Genes Dev. 16, 870–879. 10.1101/gad.965702 11937494 PMC186331

[B60] Oliveira M. C. Vullings J. van de Loo F. A. J. (2020). Osteoporosis and Osteoarthritis Are Two Sides of the Same coin Paid for Obesity. Nutrition 70, 110486. 10.1016/j.nut.2019.04.001 31655472

[B61] Onuora S. (2021). Sprifermin Benefits Maintained at 5 Years. Nat. Rev. Rheumatol. 17, 378. 10.1038/s41584-021-00643-w 34075240

[B62] Park Y.-C. Goo B.-H. Park K.-J. Kim J.-Y. Baek Y.-H. (2021). Traditional Korean Medicine as Collaborating Treatments with Conventional Treatments for Knee Osteoarthritis: A Protocol for a Systematic Review and Meta-Analysis. Jpr Vol. 14, 1345–1351. 10.2147/jpr.S311557 PMC8153204 34054306

[B63] Peng Z. Sun H. Bunpetch V. Koh Y. Wen Y. Wu D. (2021). The Regulation of Cartilage Extracellular Matrix Homeostasis in Joint Cartilage Degeneration and Regeneration. Biomaterials 268, 120555. 10.1016/j.biomaterials.2020.120555 33285440

[B64] Power J. Hernandez P. Guehring H. Getgood A. Henson F. (2014). Intra-articular Injection of rhFGF-18 Improves the Healing in Microfracture Treated Chondral Defects in an Ovine Model. J. Orthop. Res. 32, 669–676. 10.1002/jor.22580 24436147

[B65] Pu P. Qingyuan M. Weishan W. Fei H. Tengyang M. Weiping Z. (2021). Protein-Degrading Enzymes in Osteoarthritis. Z. Orthop. Unfall 159, 54–66. 10.1055/a-1019-8117 31746442

[B66] Quinn R. H. Murray J. N. Pezold R. Sevarino K. S. (2018). Surgical Management of Osteoarthritis of the Knee. J. Am. Acad. Orthopaedic Surgeons 26, e191–e193. 10.5435/jaaos-d-17-00424 29688919

[B67] Redondo M. Beer A. Yanke A. (2018). Cartilage Restoration: Microfracture and Osteochondral Autograft Transplantation. J. Knee Surg. 31, 231–238. 10.1055/s-0037-1618592 29396963

[B68] Reker D. Kjelgaard-Petersen C. F. Siebuhr A. S. Michaelis M. Gigout A. Karsdal M. A. (2017). Sprifermin (rhFGF18) Modulates Extracellular Matrix Turnover in Cartilage Explants *Ex Vivo* . J. Transl Med. 15, 250. 10.1186/s12967-017-1356-8 29233174 PMC5727954

[B69] Reker D. Siebuhr A. S. Thudium C. S. Gantzel T. Ladel C. Michaelis M. (2020). Sprifermin (rhFGF18) versus Vehicle Induces a Biphasic Process of Extracellular Matrix Remodeling in Human Knee OA Articular Cartilage *Ex Vivo* . Sci. Rep. 10, 6011. 10.1038/s41598-020-63216-z 32265494 PMC7138815

[B70] Rim Y. A. Nam Y. Ju J. H. (2020). The Role of Chondrocyte Hypertrophy and Senescence in Osteoarthritis Initiation and Progression. Ijms 21, 2358. 10.3390/ijms21072358 PMC7177949 32235300

[B71] Ritter S. Y. Subbaiah R. Bebek G. Crish J. Scanzello C. R. Krastins B. (2013). Proteomic Analysis of Synovial Fluid from the Osteoarthritic Knee: Comparison with Transcriptome Analyses of Joint Tissues. Arthritis Rheum. 65, 981–992. 10.1002/art.37823 23400684 PMC3618606

[B72] Roemer F. W. Aydemir A. Lohmander S. Crema M. D. Marra M. D. Muurahainen N. (2016). Structural Effects of Sprifermin in Knee Osteoarthritis: a post-hoc Analysis on Cartilage and Non-cartilaginous Tissue Alterations in a Randomized Controlled Trial. BMC Musculoskelet. Disord. 17, 267. 10.1186/s12891-016-1128-2 27393009 PMC4938999

[B73] Roemer F. W. Kraines J. Aydemir A. Wax S. Hochberg M. C. Crema M. D. (2020). Evaluating the Structural Effects of Intra-articular Sprifermin on Cartilage and Non-cartilaginous Tissue Alterations, Based on sqMRI Assessment over 2 Years. Osteoarthritis and Cartilage 28, 1229–1234. 10.1016/j.joca.2020.05.015 32619609

[B74] Rozenblatt-Rosen O. Mosonego-Ornan E. Sadot E. Madar-Shapiro L. Sheinin Y. Ginsberg D. (2002). Induction of Chondrocyte Growth Arrest by FGF: Transcriptional and Cytoskeletal Alterations. J. Cel Sci 115, 553–562. 10.1242/jcs.115.3.553 11861762

[B75] Sandell L. J. Aigner T. (2001). Articular Cartilage and Changes in Arthritis: Cell Biology of Osteoarthritis. Arthritis Res. Ther. 3, 107–113. 10.1186/ar148 PMC128887 11178118

[B76] Sandya S. Achan M. A. Sudhakaran P. R. (2007). Parallel Changes in Fibronectin and Alpha5beta1 Integrin in Articular Cartilage in Type II Collagen-Induced Arthritis. Indian J. Biochem. Biophys. 44, 14–18. 17385335

[B77] Sennett M. L. Meloni G. R. Farran A. J. E. Guehring H. Mauck R. L. Dodge G. R. (2018). Sprifermin Treatment Enhances Cartilage Integration in an *In Vitro* Repair Model. J. Orthop. Res. 36, 2648–2656. 10.1002/jor.24048 29761549 PMC7241943

[B78] Shah S. S. Mithoefer K. (2020b). Current Applications of Growth Factors for Knee Cartilage Repair and Osteoarthritis Treatment. Curr. Rev. Musculoskelet. Med. 13, 641–650. 10.1007/s12178-020-09664-6 32710292 PMC7661573

[B79] Shah S. S. Mithoefer K. (2020a). Scientific Developments and Clinical Applications Utilizing Chondrons and Chondrocytes with Matrix for Cartilage Repair. Cartilage 6, 194760352096888. 10.1177/1947603520968884 PMC8808934 33155482

[B80] Shen L. Zhou L. Xia M. Lin N. Ma J. Dong D. (2021). PGC1α Regulates Mitochondrial Oxidative Phosphorylation Involved in Cisplatin Resistance in Ovarian Cancer Cells via Nucleo-Mitochondrial Transcriptional Feedback. Exp. Cel Res. 398, 112369. 10.1016/j.yexcr.2020.112369 33220258

[B81] Sherwood J. C. Bertrand J. Eldridge S. E. Dell’Accio F. (2014). Cellular and Molecular Mechanisms of Cartilage Damage and Repair. Drug Discov. Today 19, 1172–1177. 10.1016/j.drudis.2014.05.014 24880104

[B82] Sieber S. Gigout A. (2020). Sprifermin (Recombinant Human FGF18) Is Internalized through Clathrin- and Dynamin-independent Pathways and Degraded in Primary Chondrocytes. Exp. Cel Res. 395, 112236. 10.1016/j.yexcr.2020.112236 32798495

[B83] Skou S. T. Pedersen B. K. Abbott J. H. Patterson B. Barton C. (2018). Physical Activity and Exercise Therapy Benefit More Than Just Symptoms and Impairments in People with Hip and Knee Osteoarthritis. J. Orthop. Sports Phys. Ther. 48, 439–447. 10.2519/jospt.2018.7877 29669488

[B84] Springer B. D. Carter J. T. McLawhorn A. S. Scharf K. Roslin M. Kallies K. J. (2017). Obesity and the Role of Bariatric Surgery in the Surgical Management of Osteoarthritis of the Hip and Knee: a Review of the Literature. Surg. Obes. Relat. Dis. 13, 111–118. 10.1016/j.soard.2016.09.011 27865814

[B85] van den Berg W. B. (2011). Osteoarthritis Year 2010 in Review: Pathomechanisms. Osteoarthritis and Cartilage 19, 338–341. 10.1016/j.joca.2011.01.022 21324370

[B86] Varady N. H. Grodzinsky A. J. (2016). Osteoarthritis Year in Review 2015: Mechanics. Osteoarthritis and Cartilage 24, 27–35. 10.1016/j.joca.2015.08.018 26707990 PMC4693146

[B87] Vincent T. L. Wann A. K. T. (2019). Mechanoadaptation: Articular Cartilage through Thick and Thin. J. Physiol. 597, 1271–1281. 10.1113/jp275451 29917242 PMC6395418

[B88] Walsh N. E. Pearson J. Healey E. L. (2017). Physiotherapy Management of Lower Limb Osteoarthritis. Br. Med. Bull. 122, 151–161. 10.1093/bmb/ldx012 28472246

[B89] Wang J. Li J. Song D. Ni J. Ding M. Huang J. (2020a). AMPK: Implications in Osteoarthritis and Therapeutic Targets. Am. J. Transl Res. 12, 7670–7681. 33437352 PMC7791500

[B90] Wang S. Wang L. Wu C. Sun S. Pan J.-h. (2018). E2F2 Directly Regulates the STAT1 and PI3K/AKT/NF-κB Pathways to Exacerbate the Inflammatory Phenotype in Rheumatoid Arthritis Synovial Fibroblasts and Mouse Embryonic Fibroblasts. Arthritis Res. Ther. 20, 225. 10.1186/s13075-018-1713-x 30286793 PMC6235203

[B91] Wang W. Han X. Zhao T. Zhang X. Qu P. Zhao H. (2020b). AGT, Targeted by miR-149-5p, Promotes IL-6-induced Inflammatory Responses of Chondrocytes in Osteoarthritis via Activating JAK2/STAT3 Pathway. Clin. Exp. Rheumatol. 38, 1088–1095. 32141427

[B92] Whitmore T. E. Maurer M. F. Sexson S. Raymond F. Conklin D. Deisher T. A. (2000). Assignment1 of Fibroblast Growth Factor 18 (FGF18) to Human Chromosome 5q34 by Use of Radiation Hybrid Mapping and Fluorescence *In Situ* Hybridization. Cytogenet. Genome Res. 90, 231–233. 10.1159/000056775 11124520

[B93] Wolff D. G. Christophersen C. Brown S. M. Mulcahey M. K. (2021). Topical Nonsteroidal Anti-inflammatory Drugs in the Treatment of Knee Osteoarthritis: a Systematic Review and Meta-Analysis. The Physician and Sportsmedicine 49, 381–391. 10.1080/00913847.2021.1886573 33554694

[B94] Wu H. Xu T. Chen Z. Wang Y. Li K. Chen P. S. (2020). Specific Inhibition of FAK Signaling Attenuates Subchondral Bone Deterioration and Articular Cartilage Degeneration during Osteoarthritis Pathogenesis. J. Cel Physiol 235, 8653–8666. 10.1002/jcp.29709 32324278

[B95] Xie W.-q. Chen S.-f. Tao X.-h. Zhang L.-y. Hu P.-w. Pan W.-l. (2021). Melatonin: Effects on Cartilage Homeostasis and Therapeutic Prospects in Cartilage-Related Diseases. Aging Dis. 12, 297–307. 10.14336/ad.2020.0519 33532142 PMC7801270

[B96] Xu C. Qu P. Deng T. Bell K. Chen J. (2019). Does Simultaneous Bilateral Total Joint Arthroplasty Increase Deep Infection Risk Compared to Staged Surgeries? A Meta-Analysis. J. Hosp. Infect. 101, 214–221. 10.1016/j.jhin.2018.08.019 30194025

[B97] Xu J. Yi Y. Li L. Zhang W. Wang J. (2015). Osteopontin Induces Vascular Endothelial Growth Factor Expression in Articular Cartilage through PI3K/AKT and ERK1/2 Signaling. Mol. Med. Rep. 12, 4708–4712. 10.3892/mmr.2015.3975 26099282

[B98] Xue J.-F. Shi Z.-M. Zou J. Li X.-L. (2017). Inhibition of PI3K/AKT/mTOR Signaling Pathway Promotes Autophagy of Articular Chondrocytes and Attenuates Inflammatory Response in Rats with Osteoarthritis. Biomed. Pharmacother. 89, 1252–1261. 10.1016/j.biopha.2017.01.130 28320092

[B99] Yang C.-Y. Chanalaris A. Troeberg L. (2017). ADAMTS and ADAM Metalloproteinases in Osteoarthritis - Looking beyond the 'usual Suspects'. Osteoarthritis and Cartilage 25, 1000–1009. 10.1016/j.joca.2017.02.791 28216310 PMC5473942

[B100] Yang C. Zhang Z. Ye F. Mou Z. Chen X. Ou Y. (2020). FGF18 Inhibits Clear Cell Renal Cell Carcinoma Proliferation and Invasion via Regulating Epithelial-Mesenchymal Transition. Front. Oncol. 10, 1685. 10.3389/fonc.2020.01685 33117668 PMC7552945

[B101] Yao X. Zhang J. Jing X. Ye Y. Guo J. Sun K. (2019). Fibroblast Growth Factor 18 Exerts Anti-osteoarthritic Effects through PI3K-AKT Signaling and Mitochondrial Fusion and Fission. Pharmacol. Res. 139, 314–324. 10.1016/j.phrs.2018.09.026 30273654

[B102] Youle R. J. van der Bliek A. M. (2012). Mitochondrial Fission, Fusion, and Stress. Science 337, 1062–1065. 10.1126/science.1219855 22936770 PMC4762028

[B103] Yu D. Xu J. Liu F. Wang X. Mao Y. Zhu Z. (2016). Subchondral Bone Changes and the Impacts on Joint Pain and Articular Cartilage Degeneration in Osteoarthritis. Clin. Exp. Rheumatol. 34, 929–934. 27606839

[B104] Zeng N. Chen X.-Y. Yan Z.-P. Li J.-T. Liao T. Ni G.-X. (2021). Efficacy and Safety of Sprifermin Injection for Knee Osteoarthritis Treatment: a Meta-Analysis. Arthritis Res. Ther. 23, 107. 10.1186/s13075-021-02488-w 33836824 PMC8034149

[B105] Zhang S. Hu B. Liu W. Wang P. Lv X. Chen S. (2020a). Articular Cartilage Regeneration: The Role of Endogenous Mesenchymal Stem/progenitor Cell Recruitment and Migration. Semin. Arthritis Rheum. 50, 198–208. 10.1016/j.semarthrit.2019.11.001 31767195

[B106] Zhang S. Hu B. Liu W. Wang P. Lv X. Chen S. (2021). The Role of Structure and Function Changes of Sensory Nervous System in Intervertebral Disc-Related Low Back Pain. Osteoarthritis and Cartilage 29, 17–27. 10.1016/j.joca.2020.09.002 33007412

[B107] Zhang Y. Cai W. Han G. Zhou S. Li J. Chen M. (2020b). Panax Notoginseng Saponins Prevent Senescence and Inhibit Apoptosis by Regulating the PI3K-AKT-mTOR P-athway in O-steoarthritic C-hondrocytes. Int. J. Mol. Med. 45, 1225–1236. 10.3892/ijmm.2020.4491 32124939

[B108] Zhang Z. Huang C. Jiang Q. Zheng Y. Liu Y. Liu S. (2020c). Guidelines for the Diagnosis and Treatment of Osteoarthritis in China (2019 Edition). Ann. Transl Med. 8, 1213. 10.21037/atm-20-4665 33178745 PMC7607097

[B109] Zhang Z. Li L. Yang W. Cao Y. Shi Y. Li X. (2017). The Effects of Different Doses of IGF-1 on Cartilage and Subchondral Bone during the Repair of Full-Thickness Articular Cartilage Defects in Rabbits. Osteoarthritis and Cartilage 25, 309–320. 10.1016/j.joca.2016.09.010 27662821

[B110] Zhao Z. Dai X.-S. Wang Z.-Y. Bao Z.-Q. Guan J.-Z. (2019). MicroRNA-26a Reduces Synovial Inflammation and Cartilage Injury in Osteoarthritis of Knee Joints through Impairing the NF-Κb Signaling Pathway. Biosci. Rep. 39, BSR20182025. 10.1042/bsr20182025 30872407 PMC6454017

[B111] Zhou Y. Liu S.-q. Peng H. Yu L. He B. Zhao Q. (2015). *In Vivo* anti-apoptosis Activity of Novel Berberine-Loaded Chitosan Nanoparticles Effectively Ameliorates Osteoarthritis. Int. Immunopharmacology 28, 34–43. 10.1016/j.intimp.2015.05.014 26002585

[B112] Żylińska B. Sobczyńska-rak A. Lisiecka U. Stodolak-Zych E. Jarosz Ł. Szponder T. (2021). Structure and Pathologies of Articular Cartilage. In Vivo 35, 1355–1363. 10.21873/invivo.12388 33910813 PMC8193327

